# Unlocking oncoembryonic programs for clinical advances in cancer therapy

**DOI:** 10.1002/ctm2.70409

**Published:** 2025-07-11

**Authors:** Tosca Dalessi, Hassan Fazilaty

**Affiliations:** ^1^ Department of Molecular Life Sciences University of Zurich Zurich Switzerland

**Keywords:** cancer plasticity, developmental reprogramming, oncoembryonic programs, precision oncology, therapy resistance

1

The phenotypical similarity of cancer cells to embryonic cells was first observed in the 19th century by pathologists, namely Virchow, Lobstein and Recamier.^1^, ^2^ Recent molecular analyses have substantiated these observations, revealing common transcriptional signatures and active genetic programs—oncoembryonic programs—shared between development and cancer. We now understand that reactivation of embryonic genetic programs plays a pivotal role in both tissue repair and regeneration and the progression of chronic diseases, notably cancer.^3^ In cases of extensive tissue damage, when adult stem cells are insufficient to mediate the repair, embryonic programs are reactivated to drive regeneration. Indeed, stem cells resident in adult tissues have a lower level of proliferative and migratory potential than the analogous embryonic progenitor cells, which, in contrast, can generate entire tissues in a short time. However, coincidental mutations or chronic inflammation can disrupt these programs, resulting in cells with uncontrolled growth and invasive capacity, ultimately driving cancer progression.^3^ Underscoring the connection, cancers are often described as “wounds that do not heal”.^4^ Interpreting cancer in this way highlights how the reactivation of embryonic genetic programs can become detrimental, if chronic and malfunctioning. Studying embryonic development and cancer side‐by‐side—a concept we termed ‘oncoembryology’^3^—will pave the way towards better treatments.

Despite the long‐recognised similarity, translational approaches that leverage this relationship, focusing on the specific functions of these oncoembryonic programs, are just beginning to emerge. Studying the role of oncoembryonic programs in the cancer context can be challenging due to the intricate and continuously evolving genetic and epigenetic landscape of tumours. This complexity introduces numerous confounding factors that hamper the analysis of molecular mechanisms. In contrast, the embryo provides a more controlled context to study these mechanisms in their original settings, offering valuable insights into the oncoembryonic programs. The knowledge gained from studying molecular mechanisms in the embryo provides a blueprint for better understanding the disease, devising therapeutic interventions, and testing them in pre‐clinical settings.

However, due to the nascent stage of this research, further efforts are required to achieve full translational application. A key challenge is the development of effective drugs to block the identified oncoembryonic programs. At the nexus of genetic programs are transcription factors. Embryonic transcription factors regulate numerous downstream targets, influencing cellular functions, states and fates. Many embryonic transcription factors are upregulated in tumours and thus represent an attractive therapeutic target. Important examples of embryonic transcriptional regulators upregulated in various cancers include MYC, the SOX family, and YAP/TAZ. Targeting these embryonic programs holds significant therapeutic potential, as their expressions endow cancer cells with oncogenic features such as stemness, poor differentiation, immune suppression, drug resistance, proliferation and survival.^3^, ^5^ MYC and SOX2 are pluripotency factors that are expressed in very early stages of embryogenesis. Their overactivation correlates with stemness signatures and poor differentiation in many tumours. MYC also modifies the microenvironment by suppressing the host immune response and synergises with mutant RAS to induce inflammatory reprogramming, crucial for tumour progression. Furthermore, reactivating SOX2 expression enables several cancer types to enter a transient ‘drug‐tolerant persister’ state. These persister cells are quiescent, resistant to chemotherapy, and can reawaken to form metastases long after treatment.^1^ Unfortunately, transcription factors are challenging targets for drug development. In addition, they are often also part of complex networks where other factors can compensate for their loss of function. To circumvent these challenges, researchers will need to delineate the genetic networks regulated by these transcription factors to identify key targetable nodes. For example, although multiple efforts have been made to develop drugs to inhibit YAP/TAZ action, no drug has been found that acts directly on them. The few existing YAP/TAZ signalling inhibitors target upstream components, the interaction of YAP/TAZ with the TEADs (the primary partners required to induce gene expression) or downstream targets.^5^ A better understanding of YAP/TAZ action and the genetic network is needed to identify additional effective druggable targets. Leveraging embryonic models will likely streamline this mapping process.

Targeting oncoembryonic programs, which are typically active only during embryonic development and are not necessary for adult tissue maintenance, circumvents a major challenge for most current cancer therapies: their toxicity to healthy adult tissues (Figure 1A). A promising example of such an approach is the monoclonal antibody pamrevlumab, which targets CTGF, a downstream effector of YAP/TAZ. When combined with chemotherapy, it improves the resectability of advanced pancreatic tumours without increasing toxicity.^6^

**FIGURE 1:**
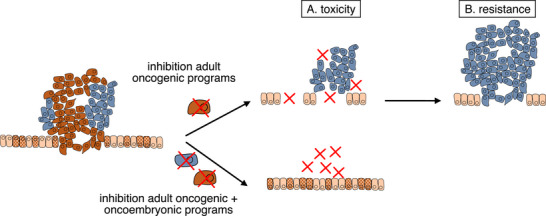
Reactivation of embryonic pathways can be used by cancer cells as a mechanism of resistance upon therapeutic targeting of adult oncogenic programs. Combination therapies are a promising approach to target cancer cell dependencies in both states. Targeting embryonic programs has the advantage of reducing toxicity for healthy adult cells, which also partially depend on the inhibited adult programs.

An additional challenge, particularly in the context of targeted therapies, is the acquisition of resistance, which impacts the majority of patients. Cancer cells have the potential to oscillate between multiple states, ranging from more adult stem‐cell‐like states to a variety of oncoembryonic states.^7^ The flux between these states can also be influenced by the therapeutic status, leading to resistance development by expanding cells in an oncoembryonic drug‐tolerant state (Figure 1B). The ability to recognise these states and having the means to target the cancer cells simultaneously from multiple angles, e.g., using combination therapies, is essential for better cancer therapy. For example, activating mutations in the receptor tyrosine kinase EGFR and downstream RAS‐MAPK signalling, which are common in solid tumours, amplify a signalling cascade active at lower levels in healthy adult cells. Targeted therapies against components of this axis are standard in the clinic. However, cancer cells often overcome their dependency on this pathway by activating alternative pathways. Multiple resistance mechanisms have been observed that lead to an overactivation of YAP/TAZ signalling, which equips cancer cells with additional growth and survival signals.^8^ Combination therapies of EGFR inhibitors and inhibitors targeting YAP/TAZ signalling are therefore being explored for their therapeutic potential.^8^

In conclusion, viewing cancer as wounds that do not heal provides a compelling framework for understanding the disease's pathology and developing innovative therapies. Integrating knowledge about the oncoembryonic programs that are activated in response to the wounds and then derailed during oncogenesis is essential to open up new therapeutic approaches. By leveraging developmental biology to investigate the function of oncoembryonic programs, researchers will be able to chart the underlying genetic networks and identify potential drug targets, as well as diagnostic markers to stratify patients. In the near future, stronger collaborative efforts between researchers and clinicians are needed to expand the integration and refine these therapies in clinical practice. This approach will allow for devising more effective treatments while avoiding resistance development and healthy tissue toxicity. Studying cancer from this perspective is a paradigm shift in our understanding of cancer biology and sets the stage for developing therapies that could transform cancer from a terminal illness into a manageable condition.

## AUTHOR CONTRIBUTIONS

Both authors contributed equally to literature review and manuscript writing.

## CONFLICT OF INTEREST STATEMENT

The authors declare no conflict of interest.

## ETHICS STATEMENT

This article does not involve original research with human participants or animals.
